# Effectiveness of a digital lifestyle management intervention (*levidex*) to improve quality of life in people with multiple sclerosis: results of a randomized controlled trial

**DOI:** 10.1186/s12883-024-03843-5

**Published:** 2024-09-16

**Authors:** Björn Meyer, Linda T. Betz, Gitta A. Jacob, Nicole Krause, Karin Riemann-Lorenz, Stefan M. Gold, Jana Pöttgen, Christoph Heesen

**Affiliations:** 1 Research and Development Department, GAIA Group, Hans-Henny-Jahnn-Weg 53, 22085 Hamburg, Germany; 2 Institute of Neuroimmunology and Multiple Sclerosis (INIMS), University Medical Center Hamburg-Eppendorf, Hamburg, Germany; 3 Medical Department, Section Psychosomatics and Department of Psychiatry and Neuroscience, Charité Universitätsmedizin Berlin, Berlin, Germany

**Keywords:** Multiple sclerosis, Digital health application, Quality of life, Randomized controlled trial, Levidex, MS symptom management

## Abstract

**Background:**

Multiple Sclerosis (MS) is a chronic inflammatory neurodegenerative disease with diverse symptomatology, significantly impacting patients’ quality of life (QoL). While pharmacological therapies focus primarily on reducing inflammation and relapse rates, non-pharmacological interventions, including digital health applications, have shown promise in improving QoL among persons with MS (PwMS). Pilot studies had shown the feasibility and acceptability of *levidex*, a digital health application based on cognitive behavioral therapy (CBT) principles, a broad set of behavior change techniques, and relevant lifestyle-change advice. This randomized controlled trial aimed to examine the effects of *levidex* on MS-related QoL over 6 months.

**Methods:**

Participants who were diagnosed with MS for at least one year were recruited via the internet in Germany, using a secure survey software platform, and were randomly assigned to the intervention group (IG), in which they received standard care + *levidex*, or an active control group (CG), in which they received standard care and were offered web-adapted material on the topic of lifestyle change from the German Multiple Sclerosis Society (DMSG). The primary outcome was MS-related QoL after 6 months, measured by the Hamburg Quality of Life Questionnaire in MS (HAQUAMS); secondary outcomes included QoL subscales, sick days, and health behavior, among others. Analyses of Covariance (ANCOVA) were used to examine intervention effects at 6 months. Participants were recruited between November 2020 and February 2022.

**Results:**

A total of 421 adult participants (mean age: 47.5, 78.1% women) were included and randomized (IG, *n* = 195, CG, *n* = 226). After 6 months, the IG exhibited significantly higher MS-related QoL, compared to the CG (total score HAQUAMS, adjusted group mean difference = -0.14, 95% CI: [-0.22, -0.06], *p* = 0.001; Cohen’s *d* = 0.23), with significant effects also observed on the cognitive and mood subscales. At 6 months, IG participants also reported significantly fewer sick days (median = 2 days in IG vs. 6 days in CG; *W* = 3939, *p* = 0.012) and significantly higher levels of daily activities, as measured by the Frenchay Activity Index, adjusted group mean difference = 1.37, 95% CI = [0.33, 2.40], *p* = 0.010; Cohen’s *d* = 0.16. Safety analyses showed no adverse events and good satisfaction.

**Conclusions:**

Compared to the control group, *levidex* facilitated clinically relevant improvements in MS-related QoL, reduced sick days, and enhanced activity in PwMS over 6 months. These findings suggest that *levidex* can serve as an effective non-pharmacological adjunctive treatment element to standard care and could help improve QoL among PwMS.

**Trial registration:**

Registered on 22.09.2020 at the German Clinical Trials Register DRKS00023023.

**Supplementary Information:**

The online version contains supplementary material available at 10.1186/s12883-024-03843-5.

## Background

Multiple sclerosis (MS) is an increasingly prevalent, chronic inflammatory neurodegenerative disease affecting the central nervous system (CNS). Globally, it impacts over 2.5 million individuals, with more than 700,000 in Europe alone, leading to considerable psychosocial burden, functional disability, and significant socio-economic costs [1, 2]. The symptomatology of MS is heterogeneous, encompassing sensory and motor impairments, fatigue, cognitive disturbances, and emotional symptomatology [3]. Current pharmacological therapies, particularly Disease-Modifying Drugs (DMDs), primarily aim to reduce inflammation and relapse rates as well as slow neurological deterioration [4]. While these treatments are mainly recommended for relapsing MS forms, some non-pharmacological interventions have shown promise in various MS forms, alleviating fatigue and depressive symptoms as well as improving quality of life (QoL) [5–7].

Recent meta-analyses have confirmed the efficacy of behavioral and psychological treatments in MS, especially those based on cognitive behavioral therapy (CBT), for improving symptoms such as fatigue, stress, and depression, and enhancing activity in daily life [8–10]. Based on emerging evidence of their relevance [11], modifications of health behaviors such as engaging in regular physical activity, adhering to a balanced diet, and smoking cessation are increasingly advocated in current MS treatment guidelines [12]. However, numerous barriers prevent many persons with MS (PwMS) from accessing effective psychological and behavioral treatments, including shortage of qualified therapists, scheduling difficulties, transportation challenges, physical impairments, fatigue, and communication problems with healthcare providers [13].

Digital health applications, which are commonly delivered via the internet (e.g., smartphones, laptops or tablet computers), could help overcome some of these access barriers, given their flexibility and ubiquity in most global regions [14]. Some digital health applications, especially those based on CBT, have shown effectiveness in reducing comorbid depressive symptoms and fatigue in PwMS [15–17]. However, research suggests that not all online interventions yield benefits, and some appear to be ineffective [15]. For example, whereas large effects on depression reduction were recently shown for an MS-specific, CBT-based digital treatment [18], another digital intervention based on problem-solving therapy did not significantly reduce depression symptoms, compared to a wait list control group [19]. Additionally, currently available digital interventions tend to focus on symptoms such as fatigue and depression, but rarely target broader behavioral self-management issues, including lifestyle and health-behavior change (e.g., diet, physical activity). This points to the need to develop and investigate more holistic MS-focused digital interventions, which, if shown to be effective, could serve as adjuncts to DMDs.

Digital interventions may be particularly appropriate for PwMS because many actively turn to the internet and eHealth technologies for information on illness management and relevant health behavior adjustments (e.g., recommended diets), indicating a gap in the provision of evidence-based patient information in typical patient-neurologist interactions [12, 16–18]. Such interventions could target modifiable risk factors, including physical activity and dietary behavior, stress management, and depression, which might help to mitigate symptom severity, improve QoL, and possibly slow disease progression [6, 10, 20, 21]. However, we are not aware of any digital intervention that targets this broad spectrum of modifiable risk factors and has been shown to improve QoL among PwMS.

In this context, we developed *levidex*, a comprehensive digital health application that is based on CBT principles, uses relevant behavior change techniques (BCTs), and conveys a broad range of evidence-based health behavior change recommendations for PwMS (see Methods section). We have previously described the program’s development, its feasibility, and preliminary data on acceptance [22, 23], showing that *levidex* was well-received by MS experts (e.g., neurologists) and PwMS. Many participants noted that the program might help users adopt a healthier diet, engage in physical activity, manage stress, and learn about other aspects of a healthy MS-specific lifestyle, which suggests that the program might facilitate improvements in QoL. However, these initial studies did not reveal whether the intervention induces long-term behavior change and improves QoL in a clinically relevant manner.

Therefore, the present randomized controlled trial (RCT) was set up, termed the DiQoLiMS trial (*Di*gital treatment to improve *Q*uality *o*f *Li*fe in *MS*). The trial aimed to examine the effectiveness of *levidex* in enhancing QoL among adult PwMS. We tested the hypothesis that the use of *levidex* in adult PwMS, in addition to their standard care, would lead to significant improvements in MS-related QoL after 6 months, compared to an active control group (CG) in which participants received standard care and were offered web-adapted material from the German Multiple Sclerosis Society (DMSG) on the topic of health behavior change in MS.

## Methods

### Study design and dates

This was a prospective parallel RCT with two arms. The study was single-blinded in the sense that study personnel was kept unaware of participants’ group assignment; however, the participants themselves could not be blinded with respect to whether they received the intervention or not. However, the unspecific factor of working through a web-based tool was controlled-for by an active CG intervention. Participants were recruited between November 2020 and February 2022.

### Setting, recruitment and data collection

Data was collected via the internet in Germany using a secure survey software platform (easyfeedback.de). After completion of the study, the data was anonymized and is stored in read-only format for ten years. Participants were recruited via online ads; that is, potentially interested participants were directed to a study webpage with detailed study information, where they could leave their contact information if they desired to participate. They were then contacted by study personnel (employees affiliated with GAIA AG), and invited to complete an online screening questionnaire to assess eligibility. It was also explained that they would have to provide written confirmation to verify their MS diagnosis (e.g., physicians’ letter).

### Randomization and allocation concealment

Randomization was performed in a 1:1 ratio (no block randomization, no stratification) using a password-protected software tool to create randomization sequences. An independent researcher developed this online randomization software tool, which has also been used in another study [24]. The allocation list stored in this randomization tool was created using a computerized random number generator programmed by the same independent researcher. The investigators of this study were blinded to this list. The allocation sequence was concealed from participants and researchers.

### Ethics approval and trial registration

The study protocol was reviewed and approved by the Ethics Committee of the Hamburg Chamber of Physicians (file number PV7375, 17.08.2020). The study was registered prospectively (DRKS00023023) and adheres to the CONSORT guidelines for reporting randomized controlled trials.

### Description of the intervention and control group

*levidex* is a complex behavioral digital health application tailored for PwMS. It was developed and is owned and operated by GAIA (www.gaia-group.com), a small-to-medium enterprise that focuses on research and development of digital health applications. The development was accompanied and supported by a multidisciplinary team of neurologists, clinical psychologists, health scientists and nutritionists, affiliated with GAIA and with the Institute of Neuroimmunology and Multiple Sclerosis (INIMS) at the University Medical Center Hamburg-Eppendorf (UKE), as described earlier [22]. Because *levidex* is a purely internet-based application and accessible on standard web-browsers, no installation of special or additional software is required. However, internet access and a current internet browser are required to use *levidex*.

The underlying software used for the development is *broca*, a proprietary software developed by GAIA, which utilizes rule-based algorithms to mimic a dialogue-like experience, such that users interact with the program by choosing from predefined response options, which are then used to custom-tailor subsequent content. The purpose of this is to simulate a supportive interaction with an empathic therapist, and to tailor information and exercises to individual user characteristics. *Broca*-based digital health applications for a range of psychiatric and somatic conditions have been shown to be effective in more than 15 RCTs [16, 17, 25–29].

To facilitate behavior change, *levidex* employs techniques gleaned from CBT (e.g., identification and refutation of unhelpful automatic thoughts, beliefs, and cognitive distortions; behavioral activation; mindfulness and relaxation exercises) as well as BCTs described in health psychology and behavioral medicine (e.g., goal-setting, self-monitoring, action planning, providing information about health consequences, conveying the credibility of the information source, encouraging supportive self-talk, and providing prompts or cues for health-promoting behavior) [30]. Like other *broca*-based interventions, *levidex* also includes mental imagery and mindfulness/acceptance exercises, both as audio recordings and in text form.

*levidex* consists of 16 content modules or “conversations”, which convey information on four broad topics: (1) General education and information provision on MS self-management, (2) psychological techniques to improve emotional well-being and prevent distress or depression, (3) dietary approaches to promote immune system health and support MS management, and (4) techniques to promote regular and appropriate levels of physical activity as well as improve sleep quality. These 16 modules are offered in three program phases or clusters: (1) six introductory modules aim to build foundational knowledge and skills (modules 1–6); (2) six advanced modules aim to build upon the previous phase and facilitate integration of therapeutic techniques into daily routines (modules 7–12); and (3) four final modules aim to recapitulate and consolidate previous content and support long-term maintenance of health-promoting habits (modules 13–16). Each module takes about 30 to 45 min to complete, depending on reading speed, individual paths through the program and decisions to listen to or skip optional audio exercises. The modules include tasks to be completed outside of *levidex* (e.g. planning exercises or shopping for certain foods) as well as exercises embedded within *levidex* (e.g. mindfulness meditation audio exercises). New modules are activated successively after a waiting period, allowing participants to reflect on the content and complete tasks and exercises before starting a new module. Optional e-mails and short text messages inform participants about newly available modules. Further detail on program development, feasibility and acceptance has been reported separately [22, 23].

Participants in the CG were offered web-adapted material on the topic of relevant health behavior change from the DMSG. Like participants in the intervention group (IG), those in the CG were permitted to continue with standard care, as coordinated by their treatment teams.

### Measures and data collection

Data was collected at three time-points: baseline, 3 months, and 6 months. The primary endpoint was MS-related QoL after 6 months, as measured by the total score of the Hamburg Quality of Life Questionnaire for MS (HAQUAMS [31, 32]). Higher scores on the HAQUAMS represent more impairment, whereas lower scores represent better QoL. The latest update (version 10.0) of this MS-specific QoL instrument contains 44 items, 28 of which can be used to derive six subscales, as described below. The HAQUAMS and has been shown to have adequate psychometric properties, including internal consistency, test-retest reliability, and convergent and discriminant validity vis-à-vis other health measures; moreover, treatment responsiveness has been demonstrated in a range of clinical settings [31, 33]. Secondary endpoints included the following:

1. Well-being: measured by the World Health Organization-Five Well Being Index (WHO-5) [34];
2. Six subscales or QoL-domains of the HAQUAMS: (a) upper extremity, (b) lower extremity, (c) fatigue, (d) cognition, (e) mood, and (f) communication;
3. MS-related socioeconomic costs: days of sick leave, hospitalizations, relevant pharmacological treatment - antidepressants, DMDs (considered in total and separately by efficacy category 1–3 (according to current guideline [12]), analgesics, systemic corticosteroids, and other nervous system agents);
4. Movement/Physical activity: (a) self-reported walking ability - MS Walking Scale 12 (MSWS-12) [35]; (b) Instrumental activities of daily living - Frenchay Activity Index (FAI) [36];
5. Dietary behavior: (a) short form of the Diet Quality Screener (sDQS) [37]; (b) Food Quality Questionnaire (FQQ) [28].

User satisfaction was measured using the “net promoter score” (NPS). Participants were asked whether they would recommend the program to a friend or colleague [38]. The NPS value indicates the probability with which users would recommend a program. Responses were classified on an 11-point numerical rating scale, with 0 = “I would definitely not recommend the program” to 10 = “I would definitely recommend the program”.

### Population

People of all sexes were included in the study. Eligibility to participate was defined by the following inclusion and exclusion criteria: (1) Age 18 to 65 years; (2) confirmed diagnosis of MS for at least one year; (3) specialist medical treatment in the last three months before study inclusion, (4) provision of consent to participate in the study; (4) sufficient comprehension of the German language; (5) access to the Internet via own smartphone or computer. The diagnosis of MS had to be confirmed in an official written document (physician’s letter or equivalent) provided to the central study center. All participants received standard medical care in individual consultation with their respective treatment teams.

### Target sample size and statistical analyses

The sample size estimation for this study was performed using G*Power (Version 3.1.9.2) to detect an intervention effect of Cohen’s *d* = 0.3 with a Type I error rate (α) of 0.05 and a power (1-β) of 0.80. Assuming a two-sided t-test for independent groups with equal allocation (1:1), the required sample size was calculated to be *n* = 352 (176 participants per group). To account for a potential dropout rate of 20%, we planned to recruit a total of 422 participants.

ANCOVA was calculated for continuous outcomes to examine intervention effects at 6 months. The respective outcome after 6 months served as the dependent variable, the treatment condition (IG vs. CG) as the independent variable, and baseline values of the respective outcome were used as the covariate. Between-group effects (Cohen’s *d*) were determined based on the difference in the observed means between IG and CG after 6 months.

The primary analysis was performed as an intention-to-treat (ITT) analysis with multiple imputation under a ‘missing at random’ (MAR) assumption. In addition, a conservative sensitivity analysis based on reference-based multiple imputation (J2R imputation) and a complete-case analysis were calculated [39, 40]. In the ITT analysis, the missing data points at the 6-month time point were imputed using the respective variable values at the baseline and 3-month time points as well as the group membership and other sociodemographic and clinical variables (age, sex, MS progression, psychotherapy status, antidepressant use at baseline). The ITT analysis was implemented following a computationally efficient implementation for bootstrapped maximum likelihood multiple imputation by von Hippel and Bartlett (2021) [41] using the R packages bootImpute [41] and mice [42]. Specifically, 1,000 bootstrap samples of the incomplete data set (with the above-mentioned variables) were generated for each outcome variable and then the relevant outcome variable was imputed twice with the mice package with default settings (i.e. using the ‘predictive mean matching’ method with a pool of 5 candidate values from which random samples are drawn) as recommended.

As part of a conservative sensitivity analysis, these results were compared with a J2R imputation. In the reference-based imputation, it is assumed that the patients who drop out of the IG no longer participate in the intervention and that their outcomes correspond to those of the CG from this point onwards [39]. The J2R sensitivity analysis was implemented with a computationally efficient implementation for bootstrapped maximum likelihood multiple imputation by von Hippel and Bartlett (2021) [41] using the bootImpute package in R. A total of 2,000 bootstrapped imputations (1,000 bootstrap samples with 2 imputations each, as recommended) were calculated for each of the incomplete datasets using the respective variable values at baseline and 3-month time points.

For the ITT and J2R analysis, ANCOVA was performed on each imputed data set as described above, and parameters of interest were aggregated by pooling [41, 43]. Cohen’s *d* was calculated analogously in the ITT and J2R analyses for each imputed data set and then also pooled [41, 43].

In addition to the J2R analysis, only study participants who had provided complete information at the 6-month time point were included in a further sensitivity analysis (complete case analysis, CC). The modeling strategy was identical to that of the primary analysis; however, the CC analysis naturally omits the estimation and pooling of model parameters in multiply imputed data.

For outcomes reflecting count data (e.g., days in hospital), the Mann-Whitney U test was used to test whether the number of the respective outcome at 6 months differed significantly between the IG and CG. Pharmacological treatment was compared for DMDs (total and divided into efficacy categories 1–3 according to the current guideline [12]), analgesics, systemic corticosteroids and other nervous system medications using χ2-tests. In addition, the change in medication use from these groups from baseline to 6-month follow-up was compared for the IG and CG using McNemar tests. These analyses were calculated based on complete observations; no imputation was performed.

A responder analysis was performed according to the ITT principle for the primary endpoint. As specified in the study protocol, minimal clinically important difference (MCID) was defined as a change in the total HAQUAMS score from baseline to 6-month follow-up of greater than 0.22 [33]. This MCID, which was derived using an anchor-based method, is recommended by the authors of the HAQUAMS as it reflects the difference that PwMS themselves perceive as clinically significant [33]. Following the ITT principle, the imputed outcomes were dichotomized in the 2,000 imputed data sets generated as described above and then the actual statistical analysis was performed on each data set [44]: A χ^2^ test was used to test whether these proportions differed significantly between the participants in the IG and CG. The relevant statistical parameters were then pooled [41, 43].

All results were considered statistically significant at the 5% level (two-sided). All analyses were performed with R, version 4.2.1 (2022-07-10). No correction was made for multiple testing.

## Results

### Participant flow and drop-outs

**Fig. 1:**
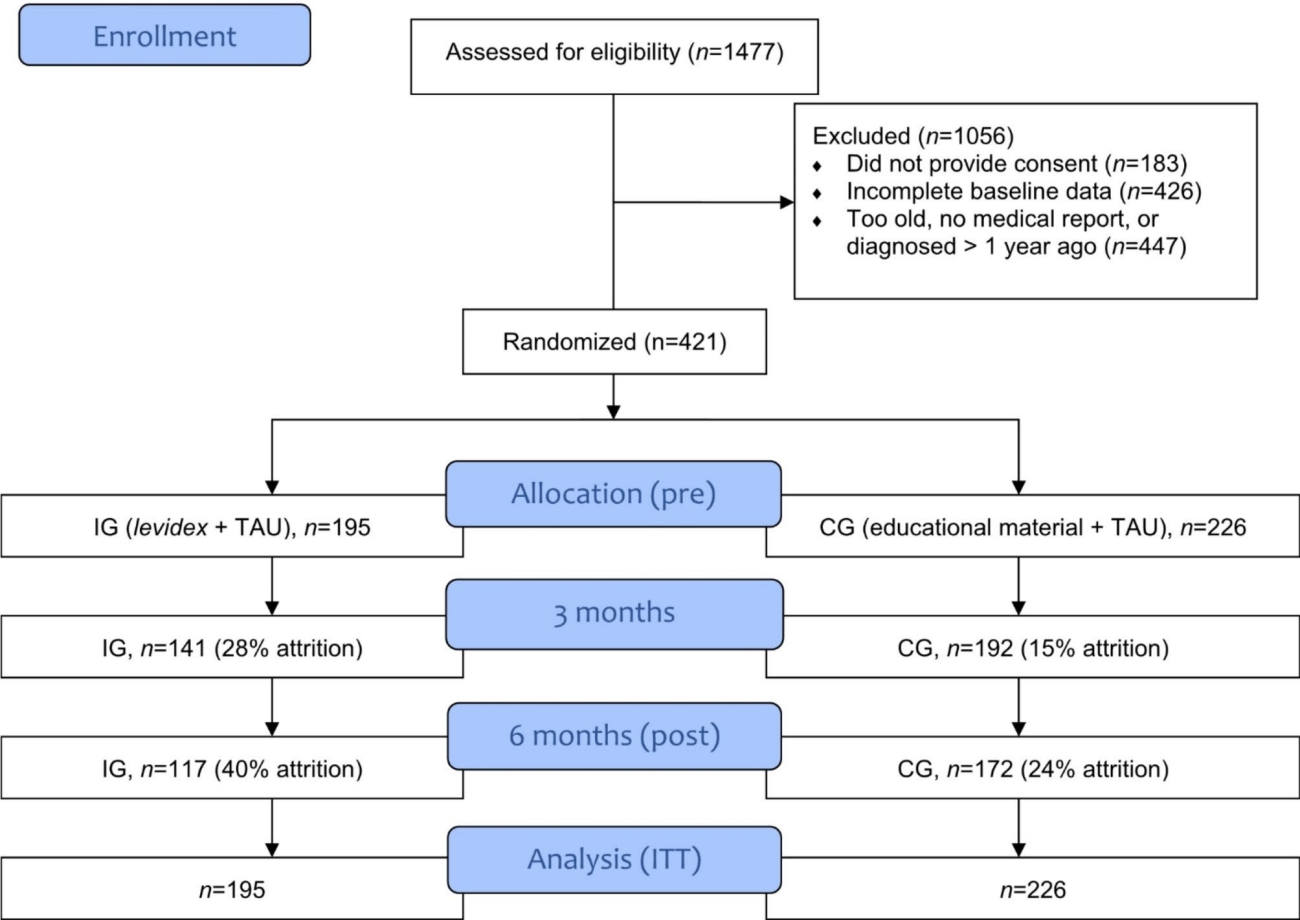
Participant flow

The study was conducted between November 2020 and March 2022. A total of 1.477 individuals initially registered to take part in the survey. Of these, 1.294 persons provided consent to participate and started the baseline online survey. Of these, 873 persons were excluded for various reasons during the screening process: (a) Age > 65 years: *n* = 72; (b) first diagnosis of MS < 1 year: *n* = 171; (c) no medical confirmation of diagnosis: *n* = 204; (d) incomplete baseline data: *n* = 426. Thus, a total of 421 participants were included in the study (see Fig. 1).

At the primary endpoint (6 months), 78 participants in the IG did not complete questionnaires (40% drop-out), compared to 54 participants in the CG (24% drop-out). Drop-outs did not differ from non-drop-outs in terms of age or sex (*p* = 0.09).

### Sociodemographic characteristics

As shown in Fig. 1, a total of 421 PwMS were randomized (*n* = 195 IG and *n* = 226 CG). Sociodemographic and clinical characteristics at baseline are shown in Tables 1 and 2.

**Table 1:** Sociodemographic characteristics

	Intervention group	Control group	Total sample
	n = 195	n = 226	N = 421
**Age**	m = 47.7	m = 47.2	m = 47.4
	(SD = 10.0,	(SD = 10.6,	(SD = 10.3,
	range = 21–65)	range = 19–65)	range = 19–65)
**Sex**			
Female	153 (78.5%)	178 (78.8%)	331 (78.6%)
Male	42 (21.5%)	48 (21.2%)	90 (21.4%)
**Education**			
Secondary school / other	59 (30.3%)	59 (26.1%)	118 (28.0%)
High school / vocational training	64 (32.8%)	83 (36.7%)	147 (34.9%)
University degree	72 (36.9%)	84 (37.2%)	156 (37.1%)
**Employment status**			
Employed full-time	58 (29.7%)	82 (36.3%)	140 (33.3%)
Employed part-time	43 (22.1%)	49 (21.7%)	92 (21.9%)
Unemployed	68 (34.9%)	80 (35.4%)	148 (35.2%)
Other	26 (13.3%)	15 (6.6%)	41 (9.7%)
**Family status**			
Married or in stable relationship	110 (56.4%)	129 (57.1%)	239 (56.8%)
Separated from partner	6 (3.1%)	8 (3.5%)	14 (3.3%)
Single	16 (8.2%)	27 (11.9%)	43 (10.2%)
In relationship, not cohabiting	41 (21.0%)	38 (16.8%)	79 (18.8%)
Divorced	19 (9.7%)	24 (10.6%)	43 (10.2%)
Widowed	3 (1.5%)	0 (0%)	3 (< 1%)
**Annual income**			
< 25.000 €	102 (52.3%)	95 (42.0%)	197 (46.8%)
25.000–50.000 €	62 (31.8%)	87 (38.5%)	149 (35.4%)
> 50.000 €	31 (15.9%)	44 (19.5%)	75 (17.8%)

Note: SD = standard deviation

**Table 2:** Clinical characteristics

			Intervention group		Control group		Total sample	
			*n* = 195		*n* = 226		*N* = 421	
**Ongoing psychotherapy**			52 (26.7%)		44 (19.5%)		96 (22.8%)	
**MS type**								
CIS			2 (1.0%)		0 (0%)		2 (< 1%)	
RRMS			97 (49.7%)		117 (51.8%)		214 (50.8%)	
PPMS			31 (15.9%)		38 (16.8%)		69 (16.4%)	
SPMS			46 (23.6%)		43 (19.0%)		89 (21.1%)	
unknown			19 (9.7%)		28 (12.4%)		47 (11.2%)	
**Medication**								
DMD total (Multiple answers possible)			103 (52.8%)		116 (51.3%)		219 (52.0%)	
Category 1^a^			58 (29.7%)		70 (31.0%)		128 (30.4%)	
Category 2^b^			16 (8.2%)		19 (8.4%)		35 (8.3%)	
Category 3^c^			31 (15.9%)		30 (13.3%)		61 (14.5%)	
Antidepressants			15 (7.7%)		19 (8.4%)		34 (8.1%)	
Analgesics			18 (9.2%)		28 (12.4%)		46 (10.9%)	
Other nervous system remedies			26 (13.3%)		26 (11.5%)		52 (12.4%)	
**Physical therapy**								
Physiotherapy			48 (24.6%)		52 (23.0%)		100 (23.8%)	
Massage			25 (12.8%)		35 (15.5%)		60 (14.3%)	

CIS: clinically isolated syndrome; RRMS: relapsing-remitting MS; PPMS: primary progressive MS; SPMS: secondary progressive MS; a: Effectiveness category 1 according to the guidelines [12] (relative reduction in relapse rate compared to placebo of 30–50%): beta interferons, dimethyl fumarate, glatirameroids and teriflunomide; b: Effectiveness category 2 according to the guidelines [12] (relative reduction in relapse rate compared to placebo of 50–60%): Cladribine, Fingolimod and Ozanimod; c: Effectiveness category 3 according to guidelines [12] (Reduction in relapse rate of > 60% compared to placebo or > 40% compared to category 1 substances): alemtuzumab, CD20 antibodies (ocrelizumab, off-label rituximab) and natalizumab. Mitoxantrone was not taken into account in the guidelines, but was assigned to efficacy category 3 in this study

### Internal consistency of questionnaires

The internal consistency of the questionnaires was assessed using Cronbach’s alpha. The HAQUAMS total score showed good internal consistency (α = 0.87). Subscales of the HAQUAMS also demonstrated acceptable to excellent internal consistency: Fatigue (α = 0.87), Cognition (α = 0.89), Lower Extremity (α = 0.91), Upper Extremity (α = 0.86), Communication (α = 0.70), and Mood (α = 0.86). Other measures used in the study also had high internal consistency: WHO-5 (α = 0.85), MSWS (α = 0.97), FAI (α = 0.84), and FQQ (α = 0.88). The sDQS showed a lower but acceptable internal consistency (α = 0.67).

### Primary endpoint

**Fig. 2:**
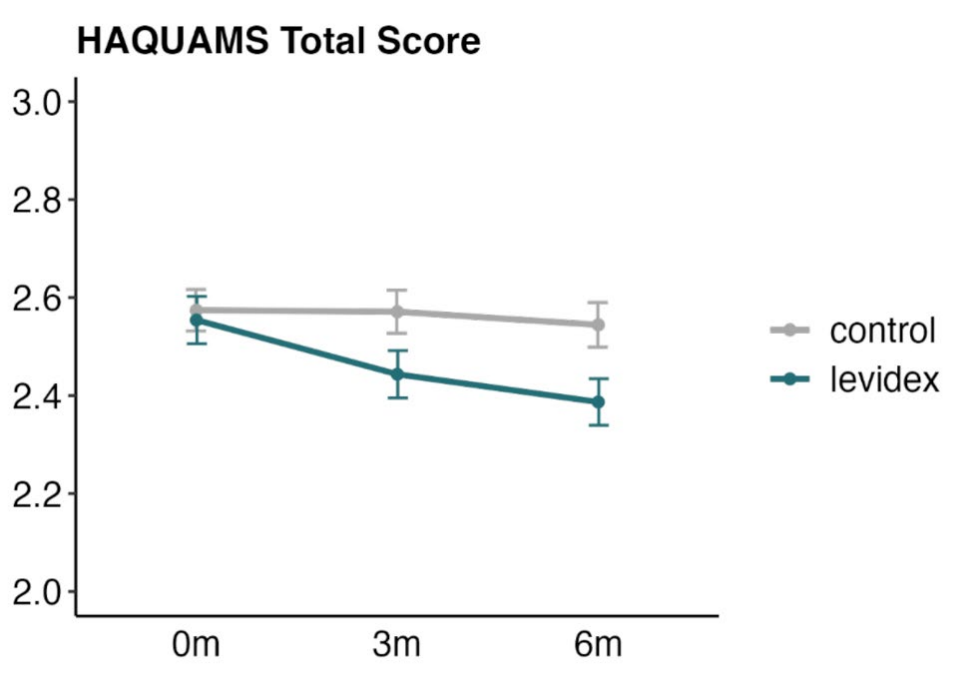
Changes in MS-related QoL (Hamburg Quality of Life in MS questionnaire, HAQUAMS total, primary endpoint at month 6). The error bars represent the standard errors of the mean

The primary analysis (ITT data) showed significant improvement in MS-related QoL in the IG compared to the CG, as measured by the HAQUAMS total score at 6 months (Table 3). This statistically significant effect was small in magnitude and was confirmed in the J2R and complete-case sensitivity analyses. The results of the ITT analysis are also presented in Fig. 2, showing that intervention effects on QoL improvements appeared to slightly increase over time.

**Table 3:** Primary endpoint: MS-related quality of life 6 months after baseline, assessed using the total score of the Hamburg Quality of Life in MS questionnaire (HAQUAMS) in ITT analyses

	Time	Control			levidex			ANCOVA				
		*n*	M	SD	*n*	M	SD	Treatment effect (95% CI)^a^	*p*-Value		Cohen’s *d* (95% CI)^b^	
ITT	pre	226	2.57	0.64	195	2.55	0.68					
	post	226	2.54	0.68	195	2.39	0.66	-0.14 (-0.22, -0.06)	0.001		0.23 (0.04, 0.43)	
J2R	pre	226	2.58	0.64	195	2.55	0.68					
	post	226	2.54	0.69	195	2.45	0.70	-0.07 (-0.12, -0.02)	0.007		0.13 (-0.06, 0.31)	
CC	pre	226	2.57	0.64	195	2.55	0.68					
	post	172	2.50	0.68	117	2.33	0.63	-0.13 (-0.22, -0.04)	0.005		0.25 (0.02, 0.49)	

^a^ Group difference on the original scale 6 months after baseline, adjusted for baseline scores

^b^ based on observed values; positive values ​​show effects in favor of the intervention group

Note: Sensitivity analyses: J2R (Jump-to-reference); CC (complete cases)

### Secondary endpoints

Descriptive statistics and detailed results of these and all other analyses concerning secondary endpoints are presented in the online supplementary material. Statistically significant intervention effects were also observed on the HAQUAMS cognition and mood subscales, and these effects were confirmed in both sensitivity analyses (J2R and CC; *p* < 0.04 in all analyses). The effect on the HAQUAMS fatigue subscale narrowly missed significance (*p* < 0.07 in the main analysis as well as both sensitivity analyses). Effects on other HAQUAMS subscales did not attain significance.

### Supplementary analyses at 3 months

Additional analyses were conducted to examine the differences in outcomes at 3 months. These results are summarized in Supplementary Table 5. Significant improvements were observed in the IG compared to the CG on the HAQUAMS total score (treatment effect = -0.11, 95% CI [-0.19, -0.04], *p* = 0.004, *d* = 0.19), WHO-5 (treatment effect = 1.35, 95% CI [0.34, 2.35], *p* = 0.009, *d* = 0.33), HAQUAMS cognition (treatment effect = -0.14, 95% CI [-0.27, -0.02], *p* = 0.025, *d* = 0.15), HAQUAMS fatigue (treatment effect = -0.16, 95% CI [-0.31, -0.02], *p* = 0.026, *d* = 0.19), and HAQUAMS mood (treatment effect = -0.21, 95% CI [-0.34, -0.08], *p* = 0.002, *d* = 0.22). No significant differences were found for HAQUAMS communication, lower extremity, upper extremity, MSWS, FAI, sDQS, or FQQ at this time point.

### Days of sick leave

Only participants who stated at baseline that they were in full-time or part-time employment (*n* = 232) were included in the analyses concerning days of sick leave. After 6 months, participants in the IG reported significantly fewer days of sick leave (median = 2 days) than those in the CG (median = 6 sick days; *W* = 3939, *p* = 0.012).

### Days of inpatient hospitalization

After 6 months, there were no significant differences between the IG and CG in the days of inpatient hospitalization (*W* = 10562, *p* = 0.477; median = 0 days of inpatient hospitalization in both groups).

### Pharmacological treatment

After 6 months, there were no significant differences between the IG and CG in pharmacological treatment (DMDs, other CNS medications, antidepressants, corticosteroids for systemic use or analgesics). Further descriptive details on pharmacological treatments received in both groups are presented in the online supplementary material.

### Movement/Physical activity and dietary behavior

For daily activities, a significant intervention effect was observed after 6 months (FAI: adjusted group mean difference = 1.37, 95% CI = [0.33, 2.40], *p* = 0.010; *d* = 0.16), but not with respect to self-reported walking ability (MSWS-12: adjusted group mean difference = -2.67, 95% CI = [-7.24, 1.90], *p* = 0.252; *d* = 0.02).

For dietary behavior, there were no significant differences between the IG and CG after 6 months, neither on the sDQS (adjusted group mean difference = -0.48, 95% CI = [-1.31, 0.35], *p* = 0.253; *d* = 0.07) nor on the FQQ (adjusted group mean difference = 0.05, 95% CI = [-0.004, 0.11], *p* = 0.070; *d* = 0.10) (for details, see online supplementary material).

### User satisfaction

Of *n* = 112 respondents, 8.9% stated that they were not satisfied after 6 months (NRS = 0–3). 21.4% indicated medium satisfaction (NRS = 4–6), and 69.6% indicated good to very good satisfaction (NRS = 7–10). The mean of 7.28 (*SD* = 2.47) indicated that the program was typically recommended by most users, on average.

### Safety analysis

None of the participants reported any adverse effects. An additional post-hoc safety analysis was carried out on complete observations examined the proportion of participants whose MS-related QoL had deteriorated compared to baseline after 6 months. Significantly fewer PwMS in the IG reported a deterioration in MS-related QoL from baseline to 6-months (37.1%), compared to the CG (52.2%; χ2 = 6.05, *p* = 0.014). With regard to clinically significant deterioration in MS-related QoL (defined by an MCID of 0.22, see [33]), there were no significant differences between the IG and CG (IG: 17.8% vs. CG: 22.5%; χ^2^ = 0.95, *p* = 0.330).

### Subsidiary analyses with threshold for impaired quality of life

Subsidiary analyses included only participants with impaired MS-related QoL [32] at baseline (HAQUAMS total score ≥ 2). This HAQUAMS cut-off roughly corresponds to a score ≥ 3 on the Expanded Disability Status Scale (EDSS), i.e., at least moderate limitations in MS (33). After applying this threshold, *N* = 333 participants with impaired MS-related QoL remained for analysis, of whom 183 were in the CG and 150 in the IG. Re-analysis was performed according to the ITT principle for the primary outcome only (Table 4). These results confirmed a statistically significant intervention effect on improvements in MS-related QoL among participants with initially impaired MS-related QoL.

**Table 4:** Subsidiary analysis (results of the primary endpoint, MS-related QoL)

	**Time**	control			*levidex*			ANCOVA		
		n	M	SD	n	M	SD	Treatment effect (95% CI)^a^	*p*-Value	Cohen’s *d* (95% CI)^b^
ITT	pre	183	2.77	0.53	150	2.80	0.55			
	post	183	2.44	0.61	150	2.57	0.62	-0.19 (-0.30, -0.09)	< 0.001	0.28 (0.04, 0.52)

^a^ Group difference on the original scale 6 months after baseline, adjusted for baseline scores

^b^ based on observed values; positive values ​​show effects in favor of the intervention group

### Responder analysis

The statistical comparison of the number of responders (defined as described in [33]) in the ITT analysis showed that clinically relevant effects on MS-related quality of life (HAQUAMS total score) were significantly more frequent in the IG than in the CG (41.6% vs. 28.0%, *p* = 0.048), which corresponds to a number-needed-to-treat (NNT) of 7.4 (95% CI: 4.4–21.7).

### Additional moderator analyses

Additional moderator analyses were performed to explore whether intervention effects differed based on (a) concurrent psychotherapy, (b) concurrent antidepressant medication treatment, (c) type of MS (RRMS, PPMS, SPMS). The interaction between these variables and treatment group (*levidex* vs. control) was not statistically significant in any case (all *p* > 0.05), indicating that none of these variables moderated the intervention effect.

## Discussion

After 6 months of using *levidex* adjunctively to usual care, participants in the IG reported significantly higher MS-related QoL than those in the CG who only received usual care augmented with educational material on the topic of MS-specific lifestyle change. Specifically, significant improvements were observed in the total HAQUAMS score and the subscales cognition and mood, with an additional trend-level effect on the fatigue subscale. These effects were confirmed both in the conservative J2R sensitivity analysis and in the complete-case analysis. The use of *levidex* also had a significant effect on days of sick leave, and a small positive effect was observed on improvements in activities of daily living.

Additionally, exploratory analyses at the 3-month time point indicated significant improvements in several outcomes for the IG compared to the CG Notably, significant effects were observed for the HAQUAMS total score and its cognition, fatigue, and mood subscales, as well as the WHO-5, suggesting that some benefits of the intervention may manifest earlier. These early effects underscore the potential need for booster sessions to maintain and enhance these initial benefits over a longer period.

In summary, this pragmatic RCT met its primary endpoint and demonstrated that *levidex* improves MS-related QoL overall, and particularly in the areas of cognition and mood. The program also has a positive effect on activities of daily living and may reduce days of sick leave. No adverse events or serious side effects were observed. The risk-benefit ratio therefore appears to be positive.

Responder analyses confirmed that there were significantly more clinically relevant improvements at 6 months (defined as described in [33]) among participants in the IG than those in the CG (41.6% vs. 28.0%), corresponding to an NNT of 7.4. Digital treatment programs such as *levidex* could thus provide convenient access to effective, evidence-based psychosocial support to PwMS, thereby narrowing a treatment gap [45]. This is relevant because various barriers make it difficult for many PwMS to access therapist-delivered psychosocial treatment [13, 45]. Evidence-based digital tools could thus augment current treatment repertoires and support PwMS not only with regard to QoL and health behavior change, but also in terms of coping with common symptoms such as fatigue and depression, as the use of digital treatment programs has been shown to reduce depressive symptomatology [16, 18] as well as fatigue [17]. The results of the present RCT suggest that *levidex*, as a broader and more comprehensive self-management digital intervention for PwMS, compared to these other programs, is also safe, well accepted, and effective at facilitating improvements in MS-related QoL.

Several limitations of the present trial ought to be noted, including the relatively high dropout rate of 40% at the 6-month time-point in the intervention arm. The reasons for this attrition were not assessed, but previous research suggests that they might include factors such as negative treatment expectancy, low perceived credibility, dissatisfaction with the intervention, time constraints, skepticism or technical difficulties with computer programs, and sufficient subjective benefit, among others [46]. However, sensitivity analyses confirmed the robustness of the intervention effect, despite this high drop-out rate. That is, even under the conservative assumption that missing data in the IG among those who dropped out corresponded to data reported by CG participants (J2R imputation [39]), significant intervention effects were confirmed on MS-related QoL, particularly in the areas of cognition and mood, as well as on participation in activities of daily living and days of sick leave.

Depending on the assumption regarding missing data, the effects in terms of improvement in MS-related QoL ranged from *d* = 0.13 to *d* = 0.25. Even though such effects are conventionally regarded as small [47], they may still facilitate substantial improvements on a population level if disseminated broadly [48]. Thus, despite the relatively high dropout rate and small effect size, these findings may support the efficacy and potential clinical utility of this intervention for improvements in MS-related QoL, even though future studies ought to use more effective measures to reduce dropout (e.g., better initial engagement, personalized motivational and reminder messages). Other limitations include the study’s reliance on self-report measures, which can be regarded as a methodological disadvantage (e.g., subjectivity of self-reports, potential recall biases), even though it also has some advantages (e.g., relevance of patient-reported outcomes, feasibility, inherently subjective nature of the QoL construct). Furthermore, the study was conducted with PwMS in Germany; future studies could examine the program’s effectiveness in different languages and regions, similar to other studies of interventions developed with the same approach [18, 49, 50]. Another limitation is that adherence to the intervention and the control treatment were not assessed; future studies should examine potential associations between adherence and outcome, as well as differences in engagement between the intervention and control conditions.

## Conclusions

This RCT demonstrated that the digital health application *levidex*, which is based on CBT principles, a broad set of BCTs, and relevant health behavior change advice, significantly improves MS-related QoL in PwMS. Compared to a control group that received relevant, web-adapted information on behavior change, the intervention enhanced cognitive and mood aspects of MS-related QoL and daily activities over 6 months while also reducing work sick days, without any adverse effects. Moreover, the intervention was well accepted and endorsed by most participants, replicating and extending earlier pilot work. In sum, these findings suggest that *levidex* could serve as a convenient and effective adjunct to standard MS care, offering a promising avenue for improving highly patient-relevant outcomes such as QoL.

## Electronic supplementary material

Below is the link to the electronic supplementary material.

Supplementary Material 1

Supplementary Material 2

## Data Availability

Quantitative data are available from the corresponding author on reasonable request.

## Abbreviations

ANCOVA: Analysis of Covariance
BCT: Behavior change technique
CBT: Cognitive behavioral therapy
CC: Complete case analysis
CG: Control group
CNS: Central nervous system
DiQoLiMS: Digital treatment to improve Quality of Life in MS
DMSG: Deutsche Multiple Sklerose Gesellschaft (German Multiple Sclerosis Society)
DMDs: Disease-modifying Drugs
EDSS: Expanded Disability Status Scale
FAI: Frenchay Activity Index
FQQ: Food Quality Questionnaire
HAQUAMS: Hamburg Quality of Life Questionnaire in Multiple Sclerosis
IG: Intervention group
ITT: Intention to treat
INIMS: Institute of Neuroimmunology and Multiple Sclerosis
J2R: Jump-to-reference
MAR: Missing at random
MCID: Minimal clinically important difference
MS: Multiple sclerosis
MSWS-12: MS Walking Scale 12
NPS: Net promoter score
PPMS: Primary progressive MS
pwMS: Persons with MS
QoL: Quality of life
RCT: Randomized controlled trial
RRMS: Relapsing-remitting MS
SD: Standard deviation
SPMS: Secondary progressive MS
UKE: University Medical Center Hamburg-Eppendorf
WHO-5: World Health Organization-Five Well Being Index
