# Supplementary material for: Effectiveness of a digital lifestyle management intervention (levidex) to improve quality of life in people with multiple sclerosis: results of a randomized controlled trial
Source: BMC Neurol. 2024 Sep 16;24:347. doi: 10.1186/s12883-024-03843-5 (PMC11404018; doi:10.1186/s12883-024-03843-5)
Supplement: Supplementary file 1 — Supplementary Material 1 [file 12883_2024_3843_MOESM1_ESM.docx]

**Supplementary material for**

**“Effectiveness of a digital lifestyle management intervention (levidex) to improve quality of life in people with multiple sclerosis: Results of a randomized controlled trial”**

Björn Meyer, Linda Betz, Gitta Jacob, Nicole Krause, Karin Riemann-Lorenz, Stefan M. Gold, Jana Pöttgen, and Christoph Heesen

### **Secondary analyses**

1. Well-being 6 months after baseline, assessed using the World Health Organization total score-Five Well Being Index (WHO-5)

Table 1 | Results of the secondary outcome well-being (WHO-5 total score).

|  | **Time** | **control** | | | ***levidex*** | | | **ANCOVA** | |  |
| --- | --- | --- | --- | --- | --- | --- | --- | --- | --- | --- |
|  |  | n | M | SD | n | M | SD | Treatment effect  (95% CI)^a^ | *p*-Value | Cohen's *d*  (95% CI)^b^ |
| ITT | pre | 226 | 10.36 | 4.78 | 195 | 11.05 | 5.31 |  |  |  |
|  | post | 226 | 11.99 | 5.20 | 195 | 13.10 | 5.65 | 0.67  (-0.42, 1.77) | 0.229 | 0.21  (-0.02, 0.44) |
| J2R | pre | 226 | 10.37 | 4.78 | 195 | 11.05 | 5.31 |  |  |  |
|  | post | 226 | 12.03 | 5.15 | 195 | 12.81 | 5.74 | 0.33  (-0.28, 0.93) | 0.294 | 0.14  (-0.02, 0.31) |
| CC | pre | 226 | 10.38 | 4.80 | 195 | 11.06 | 5.33 |  |  |  |
|  | post | 172 | 12.10 | 5.10 | 117 | 13.54 | 5.75 | 0.73  (-0.30, 1.77) | 0.165 | 0.27  (-0.03, 0.50) |

^a^ Group difference on the original scale 6 months after baseline, adjusted for baseline scores

^b^ Based on observed values; positive values ​​show effects in favor of the intervention group

1. Disease-related Functionality 6 months after baseline, assessed using scores on the functional subscales of the HAQUAMS:
   1. Cognition
   2. Communication
   3. Fatigue
   4. Mood
   5. Lower extremity
   6. Upper extremity

Table 2a | Results of the secondary endpoint HAQUAMS *Cognition.*

|  | **Time** | **control** | | | ***levidex*** | | | **ANCOVA** | |  |
| --- | --- | --- | --- | --- | --- | --- | --- | --- | --- | --- |
|  |  | n | M | SD | n | M | SD | Treatment effect^a^  (95% CI) | *p*-Value | Cohen's *d*  (95% CI)^b^ |
| ITT | pre | 226 | 2.56 | 1.03 | 195 | 2.55 | 1.09 |  |  |  |
|  | post | 226 | 2.51 | 1.07 | 195 | 2.33 | 1.05 | -0.17  (-0.32, -0.02) | 0.026 | 0.17  (-0.05, 0.39) |
| J2R | pre | 226 | 2.56 | 1.03 | 195 | 2.55 | 1.09 |  |  |  |
|  | post | 226 | 2.51 | 1.07 | 195 | 2.40 | 1.07 | -0.10  (-0.19, -0.02) | 0.018 | 0.11  (-0.07, 0.28) |
| CC | pre | 226 | 2.56 | 1.03 | 195 | 2.55 | 1.10 |  |  |  |
|  | post | 172 | 2.5 | 1.12 | 117 | 2.35 | 1.48 | -0.16  (-0.31, -0.01) | 0.036 | 0.14  (-0.10, 0.38) |

^a^ Group difference on the original scale 6 months after baseline, adjusted for baseline scores

^b^ based on observed values; positive values ​​show effects in favor of the intervention group

Table 2b | Results of the secondary endpoint HAQUAMS *Communication.*

|  | **Time** | **control** | | | ***levidex*** | | | **ANCOVA** | |  |
| --- | --- | --- | --- | --- | --- | --- | --- | --- | --- | --- |
|  |  | n | M | SD | n | M | SD | Treatment effect^a^  (95% CI) | *p*-Value | Cohen's *d*^b^  (95% CI) |
| ITT | pre | 226 | 2.42 | 0.83 | 195 | 2.39 | 0.80 |  |  |  |
|  | post | 226 | 2.42 | 0.84 | 195 | 2.28 | 0.75 | -0.12  (-0.24, 0.01) | 0.071 | 0.17  (-0.04, 0.39) |
| J2R | pre | 226 | 2.42 | 0.83 | 195 | 2.39 | 0.80 |  |  |  |
|  | post | 226 | 2.41 | 0.84 | 195 | 2.34 | 0.79 | -0.04  (-0.12, 0.04) | 0.286 | 0.08  (-0.09, 0.25) |
| CC | pre | 226 | 2.42 | 0.84 | 195 | 2.39 | 0.80 |  |  |  |
|  | post | 172 | 2.51 | 0.83 | 117 | 2.35 | 0.81 | -0.11  (-0.25, 0.02) | 0.088 | 0.19  (-0.05, 0.42) |

^a^ Group difference on the original scale 6 months after baseline, adjusted for baseline scores

^b^ based on observed values; positive values ​​show effects in favor of the intervention group

Table 2c | Results of the secondary endpoint HAQUAMS *Fatigue.*

|  | **Time** | **control** | | | ***levidex*** | | | **ANCOVA** | |  |
| --- | --- | --- | --- | --- | --- | --- | --- | --- | --- | --- |
|  |  | n | M | SD | n | M | SD | Treatment effect^a^  (95% CI) | *p*-Value | Cohen's *d*^b^  (95% CI) |
| ITT | pre | 226 | 2.95 | 1.09 | 195 | 2.90 | 1.13 |  |  |  |
|  | post | 226 | 2.85 | 1.06 | 195 | 2.65 | 1.04 | -0.16  (-0.33, 0.003) | 0.055 | 0.18  (-0.03, 0.40) |
| J2R | pre | 226 | 2.95 | 1.10 | 195 | 2.90 | 1.13 |  |  |  |
|  | post | 226 | 2.84 | 1.07 | 195 | 2.71 | 1.05 | -0.09  (-0.19, 0.002) | 0.057 | 0.12  (-0.05, 0.29 ) |
| CC | pre | 226 | 2.95 | 1.10 | 195 | 2.93 | 1.02 |  |  |  |
|  | post | 172 | 2.90 | 1.14 | 117 | 2.72 | 0.96 | -0.15  (-0.32, 0.01) | 0.068 | 0.22  (-0.01, 0.46) |

^a^ Group difference on the original scale 6 months after baseline, adjusted for baseline scores

^b^ based on observed values; positive values ​​show effects in favor of the intervention group

Table 2d | Results of the secondary endpoint HAQUAMS *Mood.*

|  | **Time** | **control** | | | ***levidex*** | | | **ANCOVA** | |  |
| --- | --- | --- | --- | --- | --- | --- | --- | --- | --- | --- |
|  |  | n | M | SD | n | M | SD | Treatment effect^a^  (95% CI) | *p*-Value | Cohen's *d*^b^  (95% CI) |
| ITT | pre | 226 | 2.73 | 0.81 | 195 | 2.76 | 0.87 |  |  |  |
|  | post | 226 | 2.68 | 0.85 | 195 | 2.48 | 0.86 | -0.22  (-0.36, -0.07) | 0.004 | 0.23  (0.02, 0.44) |
| J2R | pre | 226 | 2.74 | 0.81 | 195 | 2.77 | 0.87 |  |  |  |
|  | post | 226 | 2.66 | 0.84 | 195 | 2.58 | 0.88 | -0.11  (-0.19, -0.02) | 0.015 | 0.10  (-0.07, 0.27 ) |
| CC | pre | 226 | 2.73 | 0.81 | 195 | 2.76 | 0.87 |  |  |  |
|  | post | 172 | 2.69 | 0.84 | 117 | 2.56 | 0.89 | -0.21  (-0.36, -0.07) | 0.004 | 0.23  (-0.001, 0.47) |

^a^ Group difference on the original scale 6 months after baseline, adjusted for baseline scores

^b^ based on observed values; positive values ​​show effects in favor of the intervention group

Table 2e | Results of the secondary endpoint HAQUAMS *Lower extremity.*

|  | **Time** | **control** | | | ***levidex*** | | | **ANCOVA** | |  |
| --- | --- | --- | --- | --- | --- | --- | --- | --- | --- | --- |
|  |  | n | M | SD | n | M | SD | Treatment effect^a^  (95% CI) | *p*-Value | Cohen's *d*^b^  (95% CI) |
| HERE | pre | 226 | 2.86 | 1.24 | 195 | 2.84 | 1.22 |  |  |  |
|  | post | 226 | 2.82 | 1.27 | 195 | 2.72 | 1.27 | -0.08  (-0.20, 0.04) | 0.210 | 0.08  (-0.12, 0.28) |
| J2R | pre | 226 | 2.87 | 1.24 | 195 | 2.84 | 1.23 |  |  |  |
|  | post | 226 | 2.82 | 1.26 | 195 | 2.75 | 1.28 | -0.04  (-0.11, 0.02) | 0.198 | 0.06  (-0.13, 0.24) |
| CC | pre | 226 | 2.86 | 1.24 | 195 | 2.84 | 1.23 |  |  |  |
|  | post | 172 | 2.77 | 1.28 | 117 | 2.75 | 1.22 | -0.05  (-0.17, 0.07) | 0.411 | 0.12  (-0.11, 0.36) |

^a^ Group difference on the original scale 6 months after baseline, adjusted for baseline scores

^b^ based on observed values; positive values ​​show effects in favor of the intervention group

Table 2f ​​| Results of the secondary endpoint HAQUAMS *Upper extremity.*

|  | **Time** | **control** | | | ***levidex*** | | | **ANCOVA** | |  |
| --- | --- | --- | --- | --- | --- | --- | --- | --- | --- | --- |
|  |  | n | M | SD | n | M | SD | Treatment effect^a^  (95% CI) | *p*-Value | Cohen's *d*^b^  (95% CI) |
| ITT | pre | 226 | 1.91 | 0.95 | 195 | 1.88 | 0.89 |  |  |  |
|  | post | 226 | 1.97 | 0.98 | 195 | 1.88 | 0.89 | -0.07  (-0.17, 0.04) | 0.208 | 0.10  (-0.09, 0.29) |
| J2R | pre | 226 | 1.91 | 0.95 | 195 | 1.88 | 0.89 |  |  |  |
|  | post | 226 | 1.97 | 0.99 | 195 | 1.91 | 0.91 | -0.04  (-0.09, 0.02) | 0.241 | 0.06  (-0.11, 0.24) |
| CC | pre | 226 | 1.91 | 0.95 | 195 | 1.88 | 0.89 |  |  |  |
|  | post | 172 | 1.96 | 0.96 | 117 | 1.90 | 0.87 | -0.09  (-0.20, 0.01) | 0.078 | 0.13  (-0.11, 0.36) |

^a^ Group difference on the original scale 6 months after baseline, adjusted for baseline scores

^b^ based on observed values; positive values ​​show effects in favor of the intervention group

Table 3 | Results for the secondary endpoint *Pharmacological treatment*

|  | **Baseline (T0)** | | | |  | **After 6 months (T2)** | | | |  | **Pre-post (according to^a^)** | |
| --- | --- | --- | --- | --- | --- | --- | --- | --- | --- | --- | --- | --- |
|  | **IG** | **CG** | **In total** | **Statistics** |  | **IG** | **CG** | **In total** | **Statistics** |  | **Statistics** | |
|  | n = 195 | n = 226 | N = 421 | (IG vs. KG) |  | n = 113 | n = 169 | N = 421 | (IG vs. KG) |  | **IG**  n = 113 | **CG**  n = 169 |
| DMD total  ((Multiple answers possible) | 103 | 116 | 219 | $\chi^{2}$(1) = 0.09, p = 0.760 |  | 84 | 47 | 131 | $\chi^{2}$(1) = 1.80, p = 0.179 |  | $\chi^{2}$(1) = 1.92, p = 0.166 | $\chi^{2}$(1) = 0.04, p = 0.847 |
| Category 1^b^ | 58 | 70 | 128 | $\chi^{2}$(1) = 0.07, p = 0.784 |  | 25 | 46 | 71 | $\chi^{2}$(1) = 0.93, p = 0.334 |  | $\chi^{2}$(1) = 7.36, p = 0.007 | $\chi^{2}$(1) = 2.0, p = 0.157 |
| Category 2^c^ | 16 | 19 | 35 | $\chi^{2}$(1) = 0.01, p = 0.940 |  | 8 | 13 | 21 | $\chi^{2}$(1) = 0.04, p = 0.848 |  | $\chi^{2}$(1) = 0, p = 1 | $\chi^{2}$(1) = 0.2, p = 0.655 |
| Category 3^d^ | 31 | 30 | 61 | $\chi^{2}$(1) = 0.58, p = 0.446 |  | 14 | 27 | 41 | $\chi^{2}$(1) = 0.70, p = 0.402 |  | $\chi^{2}$(1) = 1, p = 0.317 | $\chi^{2}$(1) = 3, p = 0.083 |
| Antidepressants | 15 | 19 | 34 | $\chi^{2}$(1) = 0.07, p = 0.788 |  | 8 | 8 | 16 | $\chi^{2}$(1) = 0.70, p = 0.404 |  | $\chi^{2}$(1) = 0.67, p = 0.414 | $\chi^{2}$(1) = 3.77, p = 0.052 |
| Analgesics | 18 | 28 | 46 | $\chi^{2}$(1) = 1.07, p = 0.300 |  | 8 | 12 | 20 | $\chi^{2}$(1) = 0.001, p = 0.99 |  | $\chi^{2}$(1) = 0.14, p = 0.705 | $\chi^{2}$(1) = 3.56,p = 0.059 |
| Systemic corticosteroids | 19 | 29 | 48 | $\chi^{2}$(1) = 0.99, p = 0.320 |  | 6 | 12 | 18 | $\chi^{2}$(1) = 0.36, p = 0.547 |  | $\chi^{2}$(1) = 3.57, p = 0.059 | $\chi^{2}$(1) = 2.67, p = 0.102 |
| Other nervous system remedies | 26 | 26 | 52 | $\chi^{2}$(1) = 0.32, p = 0.570 |  | 18 | 17 | 35 | $\chi^{2}$(1) = 2.16, p = 0.142 |  | $\chi^{2}$(1) = 0.33, p = 0.564 | $\chi^{2}$(1) = 1, p = 0.317 |
|  |  |  |  |  |  |  |  |  |  |  |  |  |

a: wgd = within-group difference; b: Effectiveness category 1 according to the guidelines[(11)](https://www.zotero.org/google-docs/?lfLzGO) (relative reduction in relapse rate compared to placebo of 30-50%): beta interferons, dimethyl fumarate, glatirameroids and teriflunomide; c: Effectiveness category 2 according to the guidelines[(11)](https://www.zotero.org/google-docs/?NR5ugO) (relative reduction in relapse rate compared to placebo of 50-60%): Cladribine, Fingolimod and Ozanimod; d: Effectiveness category 3 according to guidelines[(11)](https://www.zotero.org/google-docs/?aXHVWa) (Reduction in relapse rate of > 60% compared to placebo or > 40% compared to category 1 substances): alemtuzumab, CD20 antibodies (ocrelizumab, off-label rituximab) and natalizumab. Mitoxantrone was not taken into account in the guidelines, but was assigned to efficacy category 3 in this study.

Table 4a | Results for the secondary endpoint *Self-reported walking ability* (MSWS-12)

|  | **Time** | **control** | | | ***levidex*** | | | **ANCOVA** | |  |
| --- | --- | --- | --- | --- | --- | --- | --- | --- | --- | --- |
|  |  | n | M | SD | n | M | SD | Treatment effect^a^  (95% CI) | *p*-Value | Cohen's *d*^b^  (95% CI) |
| ITT | pre | 226 | 48.28 | 36.00 | 195 | 52.32 | 33.64 |  |  |  |
|  | post | 226 | 47.42 | 35.03 | 195 | 48.00 | 33.61 | -2.67  (-7.24, 1.90) | 0.252 | 0.02  (-0.19, 0.22) |
| J2R | pre | 226 | 48.18 | 35.98 | 195 | 52.17 | 33.65 |  |  |  |
|  | post | 226 | 46.54 | 35.88 | 195 | 49.13 | 34.30 | -0.80  (-3.70, 2.10) | 0.588 | 0.07  (-0.10, 0.25) |
| CC | pre | 218 | 36.5 | 16.2 | 190 | 38.0 | 15.2 |  |  |  |
|  | post | 164 | 34.5 | 16.9 | 111 | 33.4 | 16.1 | -1.44  (-5.85, 2.98) | 0.106 | 0.08  (-0.16, 0.32) |

^a^ Group difference on the original scale 6 months after baseline, adjusted for baseline scores

^b^ based on observed values; positive values ​​show effects in favor of the intervention group

Table 4b | Results for the secondary endpoint *General activity* (FAI)

|  | **Time** | **control** | | | ***levidex*** | | | **ANCOVA** | |  |
| --- | --- | --- | --- | --- | --- | --- | --- | --- | --- | --- |
|  |  | n | M | SD | n | M | SD | Treatment effect^a^  (95% CI) | *p*-Value | Cohen's *d*^b^  (95% CI) |
| ITT | pre | 226 | 28.16 | 9.51 | 195 | 28.29 | 8.10 |  |  |  |
|  | post | 226 | 28.10 | 9.88 | 195 | 29.59 | 8.55 | 1.37  (0.33, 2.41) | 0.010 | 0.16  (-0.03, 0.36) |
| J2R | pre | 226 | 28.17 | 9.49 | 194 | 28.33 | 8.09 |  |  |  |
|  | post | 226 | 28.15 | 9.89 | 194 | 28.97 | 8.83 | 0.67  (-0.002, 1.33) | 0.051 | 0.09  (-0.09, 0.26) |
| CC | pre | 226 | 28.2 | 9.5 | 194 | 28.3 | 8.2 |  |  |  |
|  | post | 170 | 28.8 | 9.4 | 115 | 30.2 | 8.6 | 1.37  (0.29, 2.44) | 0.008 | 0.15  (-0.09, 0.38) |

^a^ Group difference on the original scale 6 months after baseline, adjusted for baseline scores

^b^ based on observed values; positive values ​​show effects in favor of the intervention group

Table 4c | Results for the secondary endpoint *Nutrition* (sDQS)

|  | **Time** | **control** | | | ***levidex*** | | | **ANCOVA** | |  |
| --- | --- | --- | --- | --- | --- | --- | --- | --- | --- | --- |
|  |  | n | M | SD | n | M | SD | Treatment effect^a^  (95% CI) | *p*-Value | Cohen's *d*^b^  (95% CI) |
| ITT | pre | 226 | 23.51 | 4.30 | 195 | 23.85 | 4.62 |  |  |  |
|  | post | 226 | 23.67 | 4.16 | 195 | 23.38 | 4.07 | -0.48  (-1.31, 0.35) | 0.253 | 0.07  (-0.16, 0.30) |
| J2R | pre | 226 | 23.50 | 4.30 | 194 | 23.85 | 4.61 |  |  |  |
|  | post | 226 | 23.63 | 4.24 | 194 | 23.56 | 4.27 | -0.28  (-0.74, 0.18) | 0.238 | 0.02  (-0.14, 0.18) |
| CC | pre | 226 | 23.5 | 4.3 | 194 | 23.9 | 4.6 |  |  |  |
|  | post | 169 | 23.7 | 4.3 | 114 | 23.8 | 3.8 | -0.43  (-1.21, 0.36) | 0.327 | 0.02  (-0.22, -0.22) |

^a^ Group difference on the original scale 6 months after baseline, adjusted for baseline scores

^b^ based on observed values; positive values ​​show effects in favor of the intervention group

Table 4d | Results for the secondary endpoint *Nutrition* (FQQ)

|  | **Time** | **control** | | | ***levidex*** | | | **ANCOVA** | |  |
| --- | --- | --- | --- | --- | --- | --- | --- | --- | --- | --- |
|  |  | n | M | SD | n | M | SD | Treatment effect^a^  (95% CI) | *p*-Value | Cohen's *d*^b^  (95% CI) |
| ITT | pre | 226 | 2.04 | 0.44 | 195 | 2.03 | 0.42 |  |  |  |
|  | post | 226 | 2.06 | 0.42 | 195 | 2.10 | 0.40 | 0.05  (-0.004, 0.11) | 0.070 | 0.10  (-0.11, 0.31) |
| J2R | pre | 226 | 2.04 | 0.44 | 195 | 2.03 | 0.42 |  |  |  |
|  | post | 226 | 2.06 | 0.41 | 195 | 2.07 | 0.40 | 0.02  (-0.01, 0.05) | 0.179 | 0.03  (-0.16, 0.21) |
| CC | pre | 226 | 2.04 | 0.44 | 195 | 2.03 | 0.42 |  |  |  |
|  | post | 169 | 2.05 | 0.41 | 114 | 2.15 | 0.38 | 0.06  (0.002, 0.11) | 0.036 | 0.26  (0.02, 0.50) |

^a^ Group difference on the original scale 6 months after baseline, adjusted for baseline scores

^b^ based on observed values; positive values ​​show effects in favor of the intervention group

Table 5 | Results for primary and secondary endpoints after 3 months in ITT analyses.

| **Outcome** | **Time** | **control** | | ***levidex*** | | **ANCOVA** | |  |  |
| --- | --- | --- | --- | --- | --- | --- | --- | --- | --- |
|  |  | M | SD | M | SD | Treatment effect  (95% CI)^a^ | *p*-Value | Cohen's *d*  (95% CI)^b^ |  |
| HAQUAMS Total | pre | 2.57 | 0.64 | 2.55 | 0.68 | - | - | - |  |
|  | post | 2.57 | 0.66 | 2.44 | 0.67 | -0.11  (-0.19, -0.04) | 0.004 | 0.19  (-0.01, 0.39) |  |
| WHO-5 | | pre | 10.36 | 4.78 | 11.05 | 5.31 | - | - | - |
|  |  | post | 11.38 | 5.14 | 13.13 | 5.53 | 1.35  (0.34, 2.35) | 0.009 | 0.33  (0.11, 0.55) |
| HAQUAMS Cognition | | pre | 2.56 | 1.03 | 2.55 | 1.09 | - | - | - |
|  |  | post | 2.51 | 1.05 | 2.36 | 1.04 | -0.14  (-0.27, -0.02) | 0.025 | 0.15  (-0.06, 0.36) |
| HAQUAMS Communication | | pre | 2.42 | 0.83 | 2.39 | 0.80 | - | - | - |
|  |  | post | 2.46 | 0.85 | 2.35 | 0.82 | -0.08  (-0.21, 0.05) | 0.244 | 0.12  (-0.09, 0.34) |
| HAQUAMS Fatigue | | pre | 2.95 | 1.09 | 2.90 | 1.13 | - | - | - |
|  |  | post | 2.92 | 1.02 | 2.72 | 1.00 | -0.16  (-0.31, -0.02) | 0.026 | 0.19  (0.004, 0.39) |
| HAQUAMS Mood | | pre | 2.73 | 0.81 | 2.76 | 0.87 | - | - | - |
|  |  | post | 2.75 | 0.85 | 2.56 | 0.88 | -0.21  (-0.34, -0.08) | 0.002 | 0.22  (0.02, 0.42) |
| HAQUAMS Lower Extremity | | pre | 2.86 | 1.24 | 2.84 | 1.22 | - | - | - |
|  |  | post | 2.80 | 1.27 | 2.75 | 1.23 | -0.03  (-0.15, 0.09) | 0.625 | 0.04  (-0.15, 0.24) |
| HAQUAMS Upper Extremity | | pre | 1.91 | 0.95 | 1.88 | 0.89 | - | - | - |
|  |  | post | 1.98 | 0.96 | 1.89 | 0.87 | -0.06  (-0.15, 0.03) | 0.201 | 0.09  (-0.10, 0.28) |
| MSWS | | pre | 48.28 | 36.00 | 52.32 | 33.64 | - | - | - |
|  |  | post | 46.67 | 36.24 | 49.48 | 34.42 | 0.44  (-4.62, 5.50) | 0.865 | 0.08  (-0.13, 0.28) |
| FAI | | pre | 28.16 | 9.51 | 28.29 | 8.10 | - | - | - |
|  |  | post | 28.01 | 10.13 | 29.07 | 8.97 | 0.92  (-0.15, 2.0) | 0.091 | 0.11  (0.08, 0.3) |
| sDQS | | pre | 23.51 | 4.30 | 23.85 | 4.62 | - | - | - |
|  |  | post | 23.44 | 4.57 | 24.21 | 4.57 | 0.55  (-0.28, 1.38) | 0.192 | 0.17  (-0.04, 0.38) |
| FQQ | | pre | 2.04 | 0.44 | 2.03 | 0.42 | - | - | - |
|  |  | post | 2.08 | 0.41 | 2.10 | 0.41 | 0.03  (-0.02, 0.09) | 0.265 | 0.05  (-0.16, 0.27) |

^a^ Group difference on the original scale 3 months after baseline, adjusted for baseline scores

^b^ based on observed values; positive values ​​show effects in favor of the intervention group
